# Anoikis in prostate cancer bone metastasis gene signatures and therapeutic implications

**DOI:** 10.3389/fonc.2024.1446894

**Published:** 2024-09-26

**Authors:** Wei Xia, Miao Ye, Bo Jiang, Gang Xu, Guancheng Xiao, Qingming Zeng, Ruohui Huang

**Affiliations:** ^1^ Department of Urology, First Affiliated Hospital of Gannan Medical University, Ganzhou, Jiangxi, China; ^2^ Breast Diagnosis and Treatment Center, First Affiliated Hospital of Gannan Medical University, Ganzhou, Jiangxi, China

**Keywords:** prostate cancer, anoikis, model, bioinformatics, metastasis

## Abstract

**Background:**

Bone metastasis from prostate cancer severely impacts patient outcomes and quality of life. Anoikis, a form of programmed cell death triggered by the loss of cell-matrix interactions, plays a critical role in cancer progression. However, its precise relationship with prostate cancer-induced bone metastasis remains unclear. This study aims to elucidate this relationship, focusing on anoikis-related gene signatures, molecular pathways, and therapeutic implications.

**Methods:**

We used the TCGA-PRAD dataset for training, with MSKCC and GSE70769 as validation cohorts. To evaluate immunotherapy efficacy, we examined IMvigor 210 and GSE91016 datasets, and GSE137829 provided single-cell insights into prostate cancer. Specific anoikis-related genes (ARGs) were identified, and Random Survival Forest analysis and multivariate Cox regression were employed to develop anoikis-linked features. The ‘clustanoikisProfilanoikis’ and ‘GSEA’ packages were used to explore potential ARG-related pathways.

**Results:**

Analyzing 553 samples from TCGA, 231 from MSKCC, 94 from GSE70769, and single-cell data from 6 prostate cancer patients (GSE137829), we constructed a prognostic model based on 9 ARGs. GSVA revealed upregulation of carcinogenic pathways, including epithelial-mesenchymal transition, E2F targets, and angiogenesis, with downregulation of metabolic pathways. Significant differences in somatic mutations were observed between cohorts, with a positive correlation between anoikis scores and tumor mutational burden (TMB). Immune landscape analysis suggested high-risk patients might benefit more from chemotherapy than immunotherapy based on their risk score. Single-cell analysis indicated overactivation of carcinogenic pathways in the high anoikis score group.

**Conclusion:**

This study elucidates the complex interplay between anoikis and bone metastasis in prostate cancer. Our findings highlight the critical role of anoikis in metastatic progression, enhancing the understanding of key biomarkers and molecular dynamics. The identified anoikis-related gene signatures and disrupted pathways offer promising avenues for predictive and therapeutic strategies in prostate cancer management.

## 1 Introduction

Prostate cancer (PCa) represents one of the most prevalent malignancies and the second leading cause of cancer-related mortality among men in Western countries (1). Since the 1990s, prostate-specific antigen (PSA) has been used as a standard test for prostate cancer (2). However, multiple studies have shown that PSA testing does not confer a significant reduction in mortality (3). PSA levels also have limited value in predicting PCa prognosis, with 27-53% of patients experiencing biochemical recurrence (BCR) after radical prostatectomy or radiation therapy (4). BCR often precedes progression to advanced castration-resistant PCa (CRPC), which carries increased risks of distant metastasis, cancer-specific mortality, and overall mortality (5). Therefore, there is an unmet need for novel prognostic biomarkers in PCa to improve risk stratification and clinical decision-making.

Anoikis occurs when tumor cells are detached from the extracellular matrix (ECM) during metastasis, which has been documented in many studies in recent years (6). Detachment from ECM causes anoikis, a specific type of apoptosis. Epithelial and endothelial cells are responsible for anoikis, which is believed to contribute to tissue homeostasis in development (7). Apoptosis prevents isolated cells from attaching to other substrates for aberrant proliferation to protect organisms (8). In the absence of anoikis, adherent cells may suspend or proliferate in an environment other than their original ECM (9). Several cancers, including breast cancer, lung cancer, gastric cancer, and esophageal cancer, have been associated with ARGs (10–13). According to the study, FAIM2 overexpression in lung cancer leads to adverse clinical outcomes, while silencing FAIM2 may decrease tumor cell viability and resistance to anoikis (14). A novel predictor of the prognosis of colorectal cancer has been identified in KLF5, a protein that regulates cell proliferation and anoikis resistance (15). Activating cancer-initiating cells in HEC-1A cells promotes esophageal cancer epithelial-mesenchymal transition (EMT), thereby inhibiting apoptosis and negatively affecting patient outcomes (16). Lee et al. demonstrated that TMPRSS4 promotes prostate cancer cells to resist anoikis, thereby improving the survival of circulating tumor cells and promoting early metastasis, and demonstrated that TMPRSS4 promotes CSC characteristics of prostate cancer by upregulating SLUG and TWIST1-induced stem cell factor SOX2 (17).

Anoikis-related genes-based prognostic indicators are rarely analyzed in prostate cancer, despite being associated with prognosis for multiple tumors. Thus, we examined the clinical outcomes of prostate cancer patients who had combined anoikis-related genes. In our study, we identified a powerful feature and validated it in two other independent databases. In addition, we integrated single-cell data to confirm that several carcinogenic pathways in the high anoikis score group were significantly overactivated.

## 2 Results

### 2.1 Consensus clustering of anoikis-related genes

**Figure 1:**
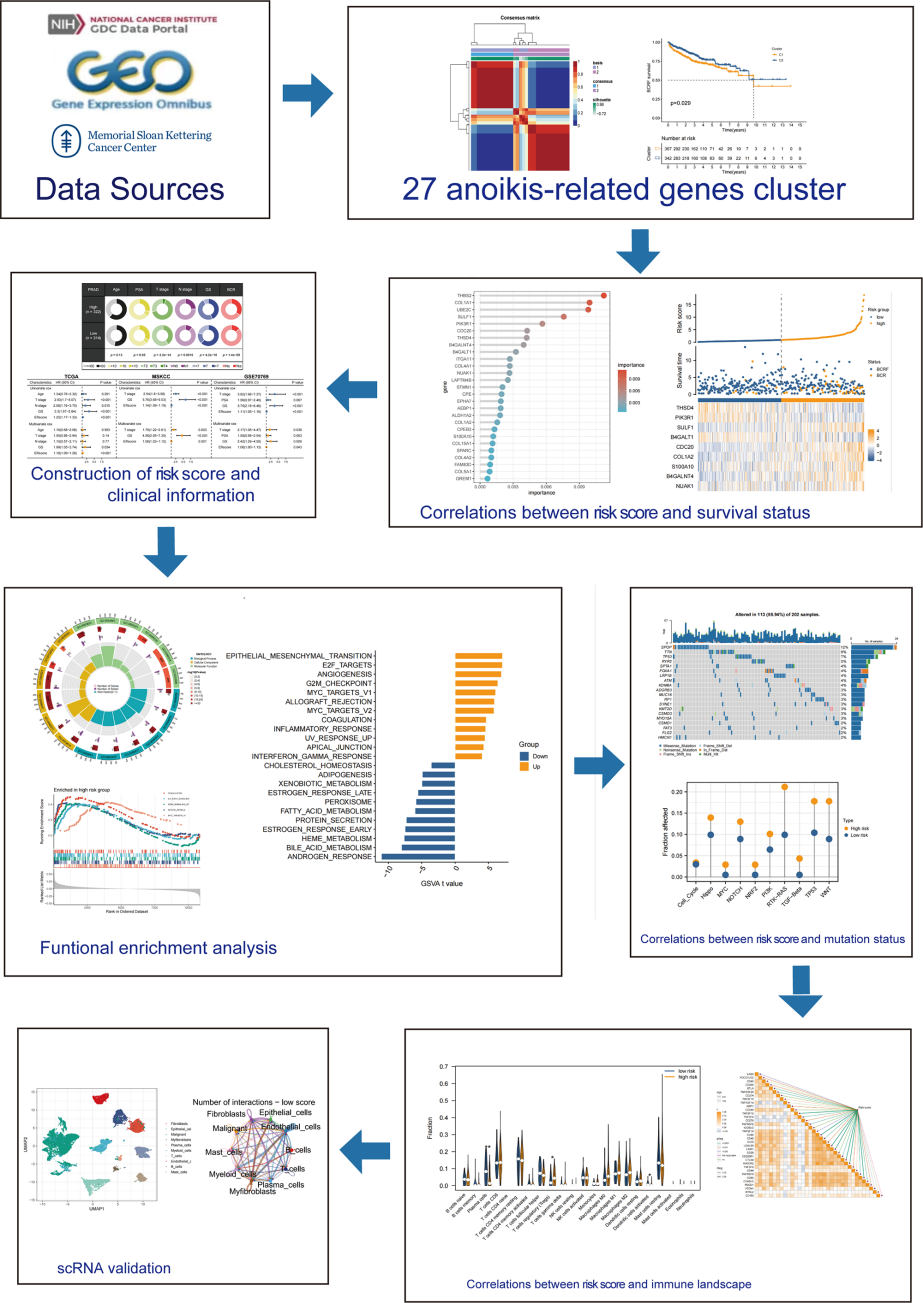
Flow chart of the main steps of this study.

**Figure 2:**
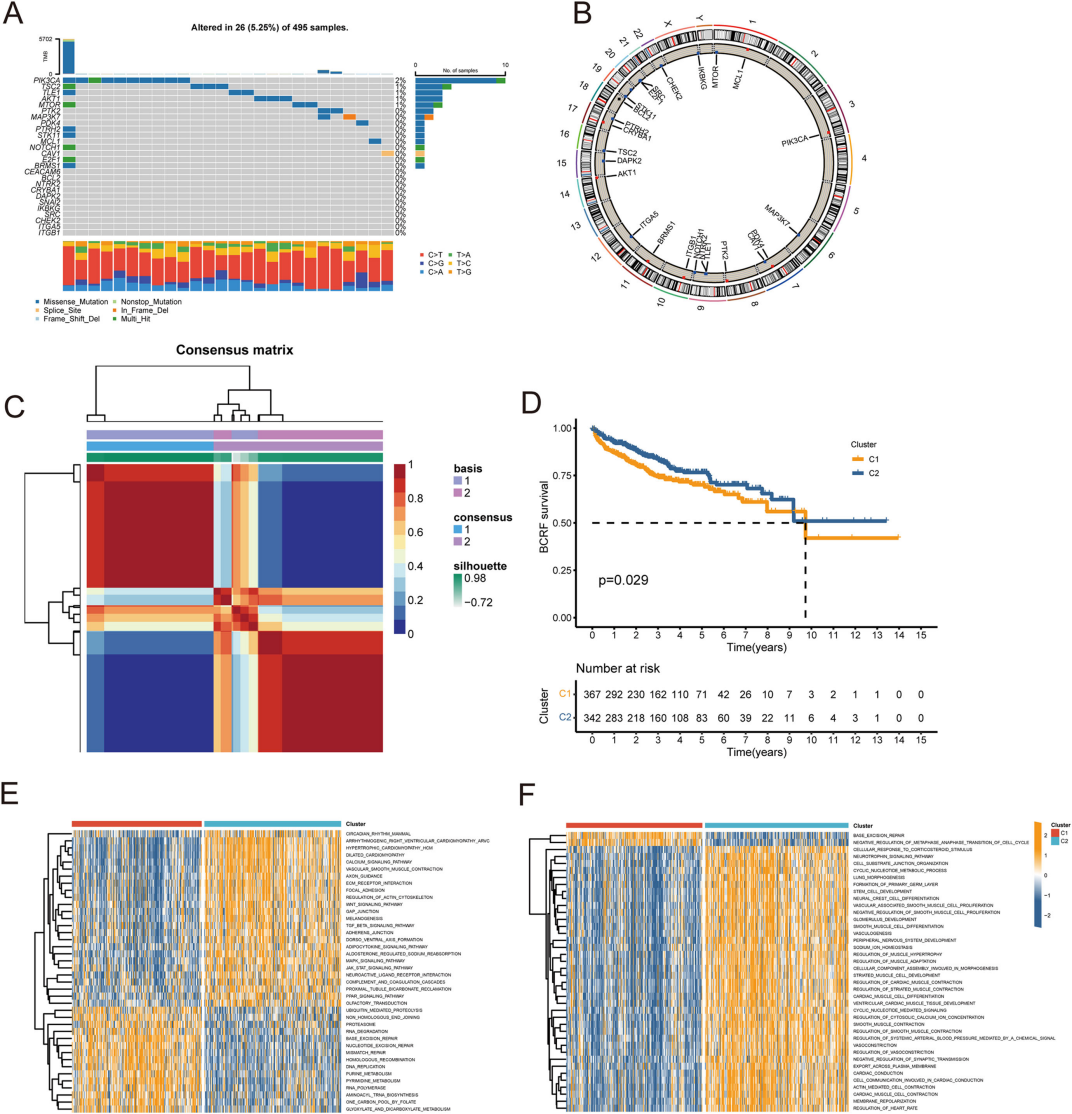
Consensus clustering of anoikis-related genes. **(A)** Waterfall plot for mutation analysis of anoikis-related genes. **(B)** Circle plot showing the location of anoikis-related genes in chromosomes. **(C)** Heatmap of NMF cluster analysis. **(D)** Survival analysis of C1 and C2 subtypes. **(E, F)** GSVA enrichment analysis of C1 and C2 Isoforms.

**Figure 1** describes the flowchart of this study. First, the mutations of anoikis related genes were analyzed. PIK3CA mutation frequency was the highest (2%), followed by TSC2, TLE1, AKT1, MTOR (**Figure 2A**). The location of anoikis-related genes in the chromosome is shown in **Figure 2B**. Accordingly, two subgroups of PRAD patients were defined based on their expression profiles of anoikis-related genes (**Figure 2C**; **Supplementary Figure S1**). K-M analysis showed that BCRF survival was significantly better in cluster 2 than in cluster 1 (**Figure 2D**). GSVA enrichment analysis showed that cluster 1 was mainly related to metabolism, mismatch repair, and cell cycle, such as NEGATIVE REGULATION OF METAPHASE ANAPHASE TRANSITION OF CELL CYCLE, BASE EXCISION REPAIR, MISMATCH REPAIR, DNA REPLICATION, GLYOXYLATE, and DICARBOXYLATE METABOLISM. PYRIMIDINE METABOLISM. On the contrary, cluster 2 is mainly related to stem cell proliferation, angiogenesis, and other pathways (**Figures 2E–F**). However, cluster 2 shows a better survival outcome, so the analysis content needs to be further explored. Taken together, our findings suggest that the two anoikis-associated subgroups are well separated in terms of prognostic outcome and biological function.

### 2.2 Anoikis-based model construction

**Figure 3:**
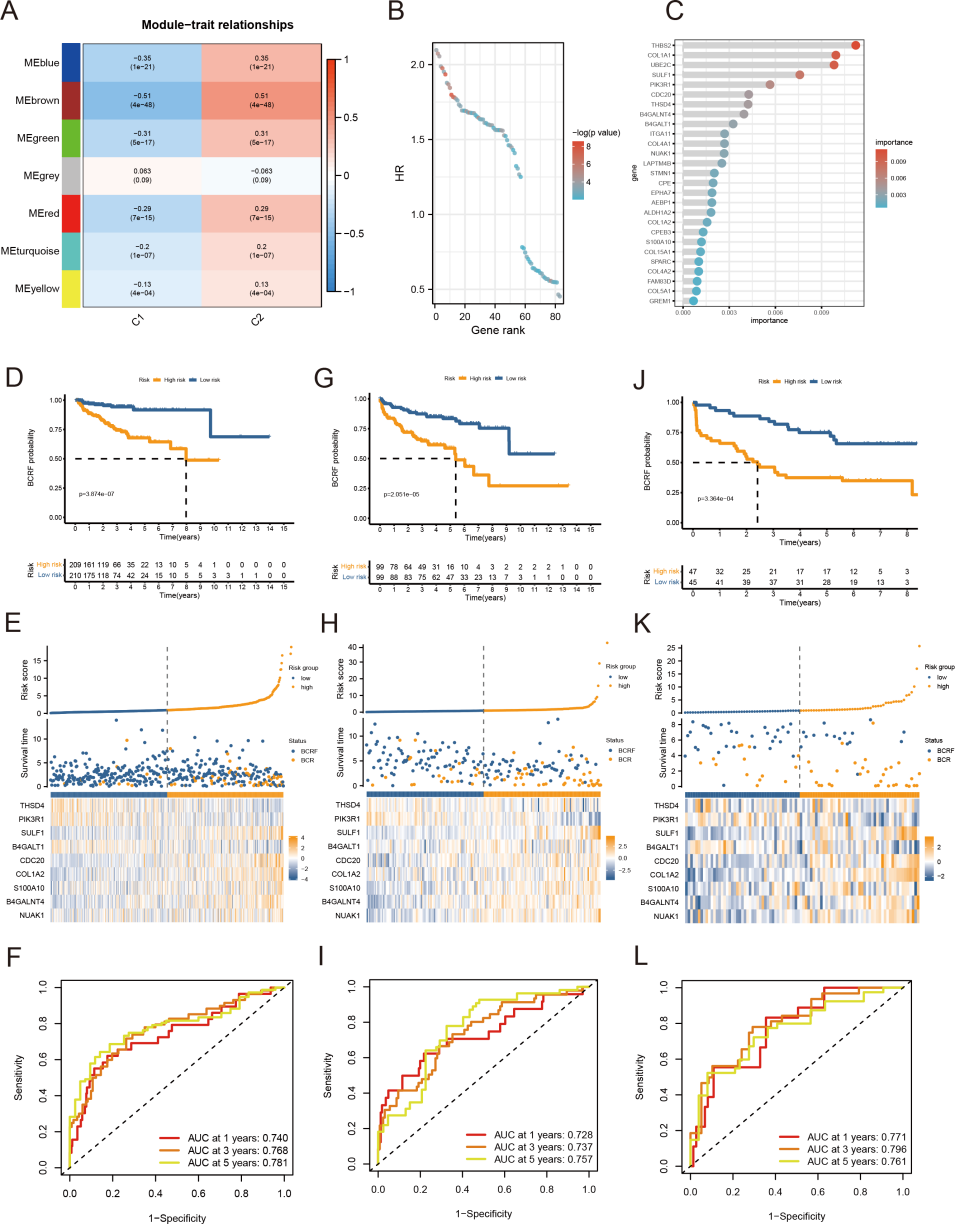
Anoikis-based model construction. **(A)** Heat map showing gene modules analyzed by WGCNA. **(B)** Dot plot for univariate Cox regression. **(C)** Screening modeling genes based on random forest analysis. **(D–F)** Kaplan – Meier curves, heat maps, and ROC curves for survival analysis of the training set cohort. **(G–I)** Kaplan – Meier curves, heat maps, and ROC curves for survival analysis of the MSKCC cohort. **(J–L)** Kaplan – Meier curves, heat maps, and ROC curves for survival analysis of the GSE70769 cohort.

As a first step, WGCNA identified the gene modules closely related to the anoikis subtype (647 genes, **Figure 3A**). The TCGA cohort was analyzed with univariate Cox regression and 83 prognostic genes were identified (**Figure 3B**). RSF analysis further identified 27 candidate genes for model construction based on the minimum depth method (**Figure 3C**). Using multivariate Cox regression, eight important genes were selected to form an anoikis score, namely THSD4, PIK3R1, SULF1, B4GALT1, CDC20, COL1A2, S100A10, B4GALNT4, NUAK1.

**Figure 4:**
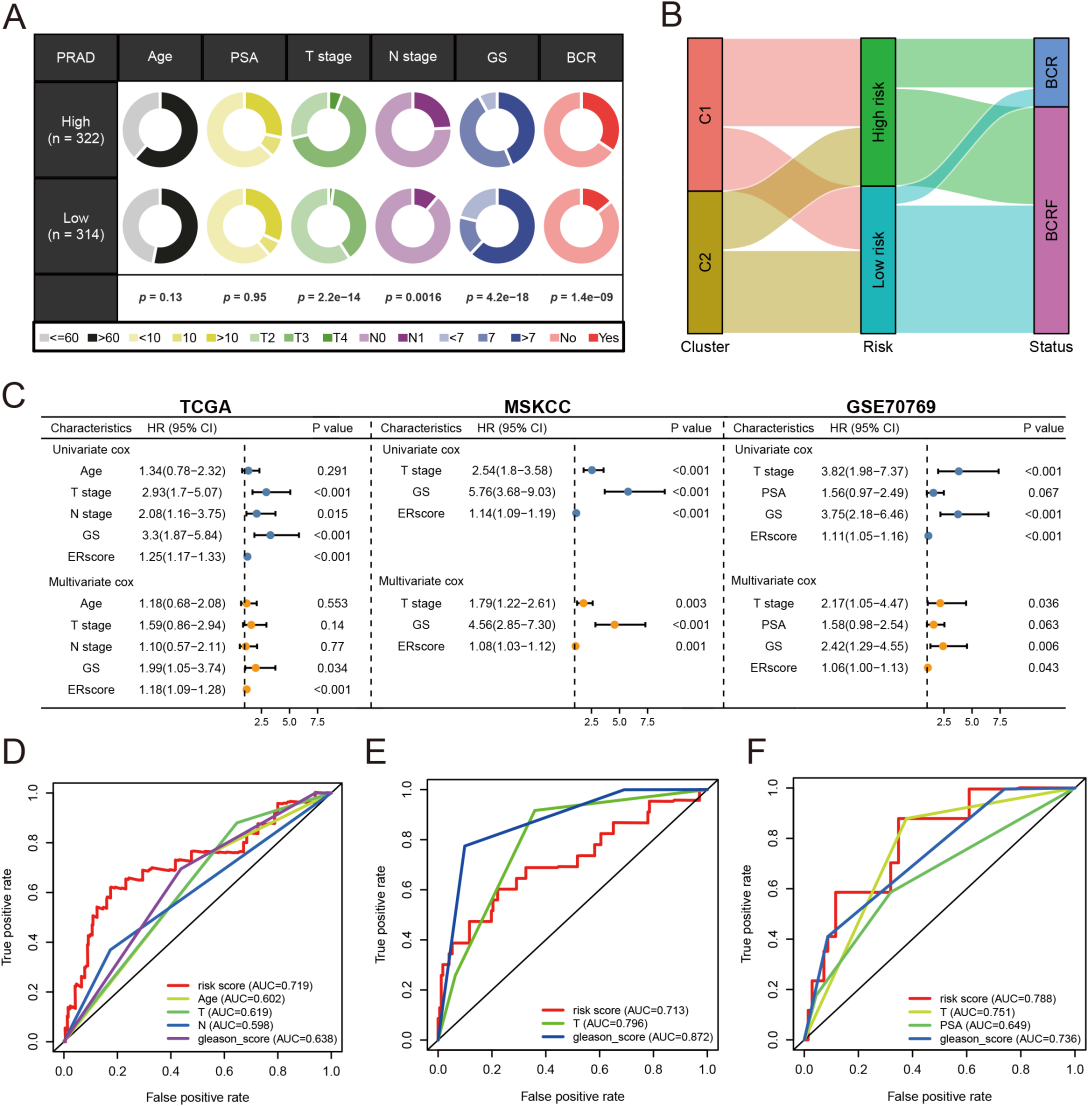
Analysis of anoikis score. **(A)** Pie chart for anoikis score versus clinical traits analysis. **(B)** Sanky chart for prognostic survival in patients at risk. **(C)** Forest plot for multivariate Cox regression analysis of risk scores in the three cohorts. **(D)** ROC analysis of anoikis score in the training set cohort. **(E)** ROC analysis of anoikis score in the MSKCC cohort. **(F)** ROC analysis of anoikis score in the GSE70769 cohort.

Patients were stratified into high-risk and low-risk groups based on the median risk score derived from the anoikis gene signature. Kaplan-Meier analysis revealed a significant difference in biochemical recurrence-free (BCRF) survival times between the high-risk and low-risk cohorts (**Figure 3D**). The distribution of risk scores, survival status, and risk level of each patient are visualized in **Figure 3E**. The anoikis gene signature demonstrated consistent prognostic power for 1-year (AUC = 0.74), 3-year (AUC = 0.768), and 5-year (AUC = 0.781) BCRF survival (**Figure 3F**). Additionally, we validated the risk model in two external datasets, MSKCC and GSE70769, where it maintained strong prognostic performance (**Figures 3G–L**). Further analysis illuminated correlations between higher anoikis scores and more advanced tumor (T) staging, higher Gleason scores (GS), and increased likelihood of BCR, implicating this gene signature as a marker of aggressive disease (**Figure 4A**). High-risk patients were also more likely to originate from the poor prognosis cluster 1 identified in our previous work (**Figure 4B**). Univariate Cox regression indicated the anoikis score and clinical variables were significantly associated with BCRF survival. Moreover, the anoikis score retained independent prognostic value in multivariate analysis after adjusting for other clinical factors (**Figure 4C**). ROC curve analysis verified the superior predictive accuracy of the risk model over individual clinical variables. External validation in the MSKCC and GSE70769 cohorts confirmed the reproducible prognostic utility of the anoikis gene signature for BCRF prediction (**Figures 4D–F**). Taken together, these findings strongly endorse the anoikis gene signature as a robust and reliable prognostic indicator for prostate cancer. Further investigation is warranted to determine the biological mechanisms underlying this model and assess its clinical value in guiding management and therapeutic decisions.

### 2.3 Functional enrichment analysis

**Figure 5:**
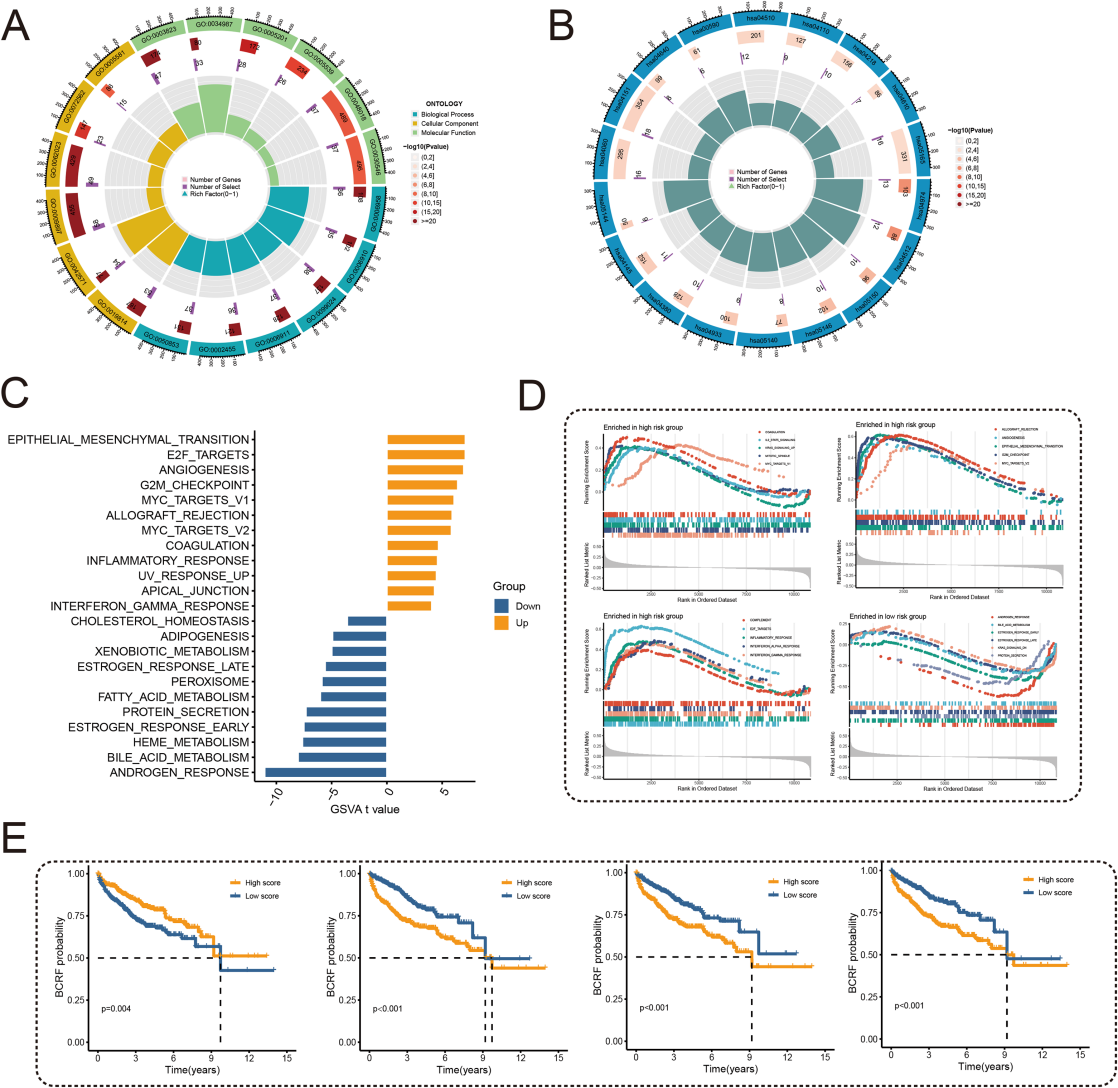
Functional enrichment analysis. **(A)** Circle plot for GO enrichment analysis. **(B)** Circle plot for KEGG enrichment analysis. **(C)** Bar graph of GSVA analysis of 50 oncogenic marker pathways. **(D)** GSEA enriched pathway analysis. **(E)** Survival curves for different BCRFs in four pathways: androgen response, E2F target, G2M check-point, MYC target V1.

To examine the potential mechanisms of risk score, GO and KEGG analyses were conducted. According to **Figure 5A**, GO analysis shows that there are mainly pathways associated with GO: 0003823, 0005201, and 0009897. According to KEGG analysis, differentially expressed genes were enriched in hsa04512, hsa05144, and other pathways (**Figure 5B**). Subsequently, 50 carcinogenic marker pathways were included in the GSVA, and the results showed that carcinogenic pathways such as EPI-THELIAL MESENCHYMAL TRANSITION, E2F TARGETS, and ANGIOGENESIS were up-regulated, while metabolic-related pathways were down-regulated (**Figure 5C**). Significant enrichment of 15 pathways was found in high-risk cohorts, whereas a significant enrichment of 5 pathways was found in low-risk cohorts, as shown in **Figure 5D**. Kaplan-Meier method was used to analyze the pathways obtained through cross-over, and different BCRF survival probabilities of several known carcinogenic path-ways (ANDROGEN RESPONSE, E2F TARGETS, G2M CHECKPOINT, MYC TARGETS V1) were observed (**Figure 5E**). Overall, the risk score is involved in a variety of biological functions, especially the carcinogenic pathways in PRAD.

### 2.4 Analysis of somatic mutations

**Figure 6:**
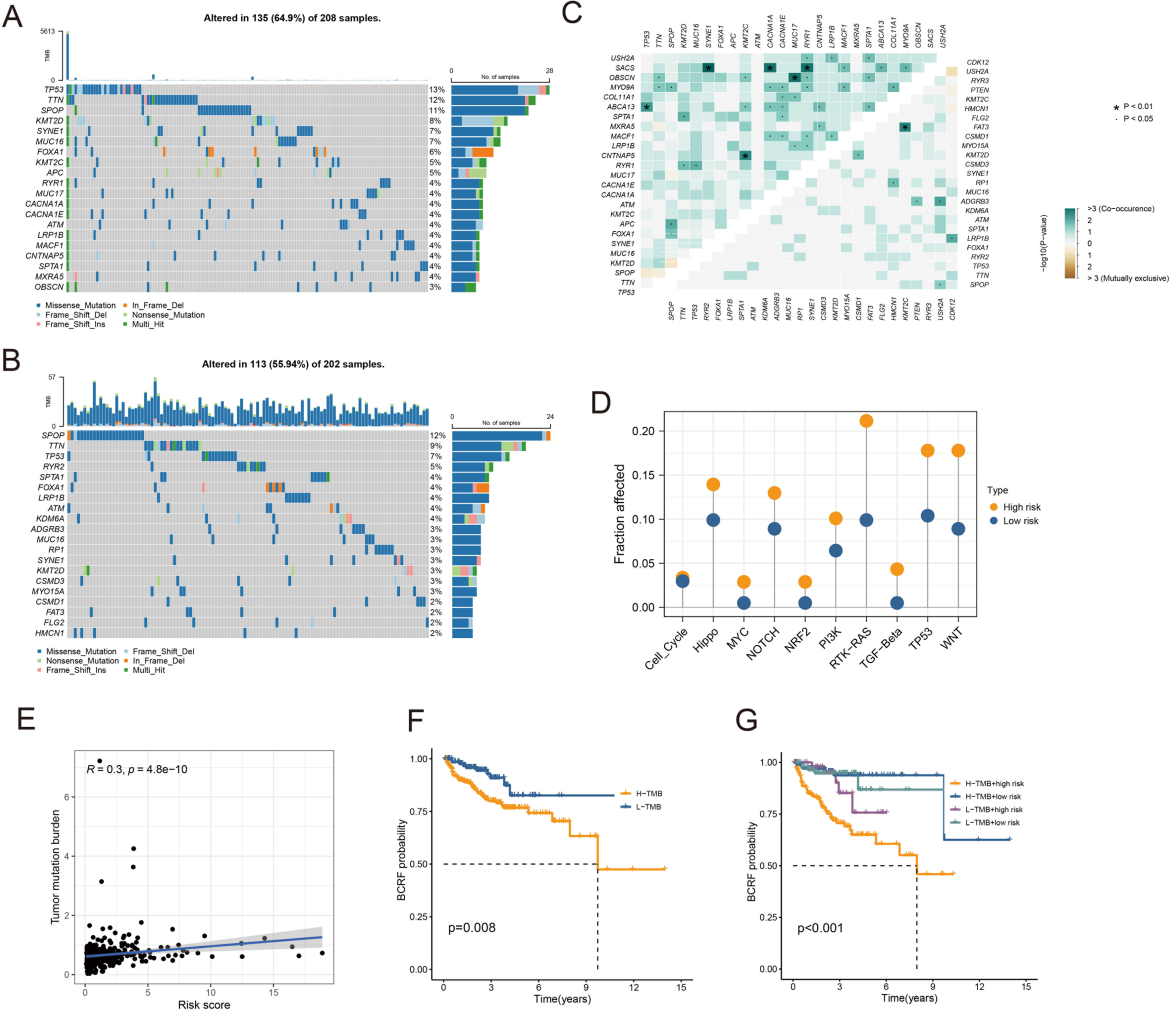
Analysis of somatic mutations. **(A)** Waterfall plot of gene mutation frequency in high-risk patients. **(B)** Waterfall plot of gene mutation frequency in low-risk patients. **(C)** Heatmap for correlation analysis between mutated genes. **(D)** Mutant gene pathway analysis between high and low-risk groups. **(E)** Scatter plot for correlation analysis between TMB and risk score. **(F)** Survival analysis between patients in high and low TMB groups. **(G)** Survival analysis between high and low TMB patients and high and low-risk patients.

As you can see in the waterfall diagram, gene mutations differ between high-risk and low-risk populations (**Figures 6A, B**). High-risk cohorts exhibited the most mutations at TP53, while low-risk cohorts showed the most mutations at SPOP. In addition, the first 25 mutant genes between the two cohorts also showed co-occurrence or exclusive mutations (**Figure 6C**). Mutation enrichment of known carcinogenic pathways showed that the Hippo, RTK-RAS, TP53, and WNT signaling pathways were significantly increased in the high-risk group, while the MYC, NRF2, and TGF-beta signaling pathways were significantly reduced (**Figure 6D**). Further analysis also confirmed a positive correlation between TMB and anoikis score, with higher TMB and poorer BCRF survival (**Figures 6F, G**). The worst prognosis was associated with high TMB and anoikis scores (**Figure 6E**). In summary, the comprehensive analysis revealed the mutational differences between high-risk and low-risk cohorts, and multiple significant genes and pathways showed significant mutation abnormalities between cohorts.

### 2.5 Immune landscape and treatment response prediction

**Figure 7:**
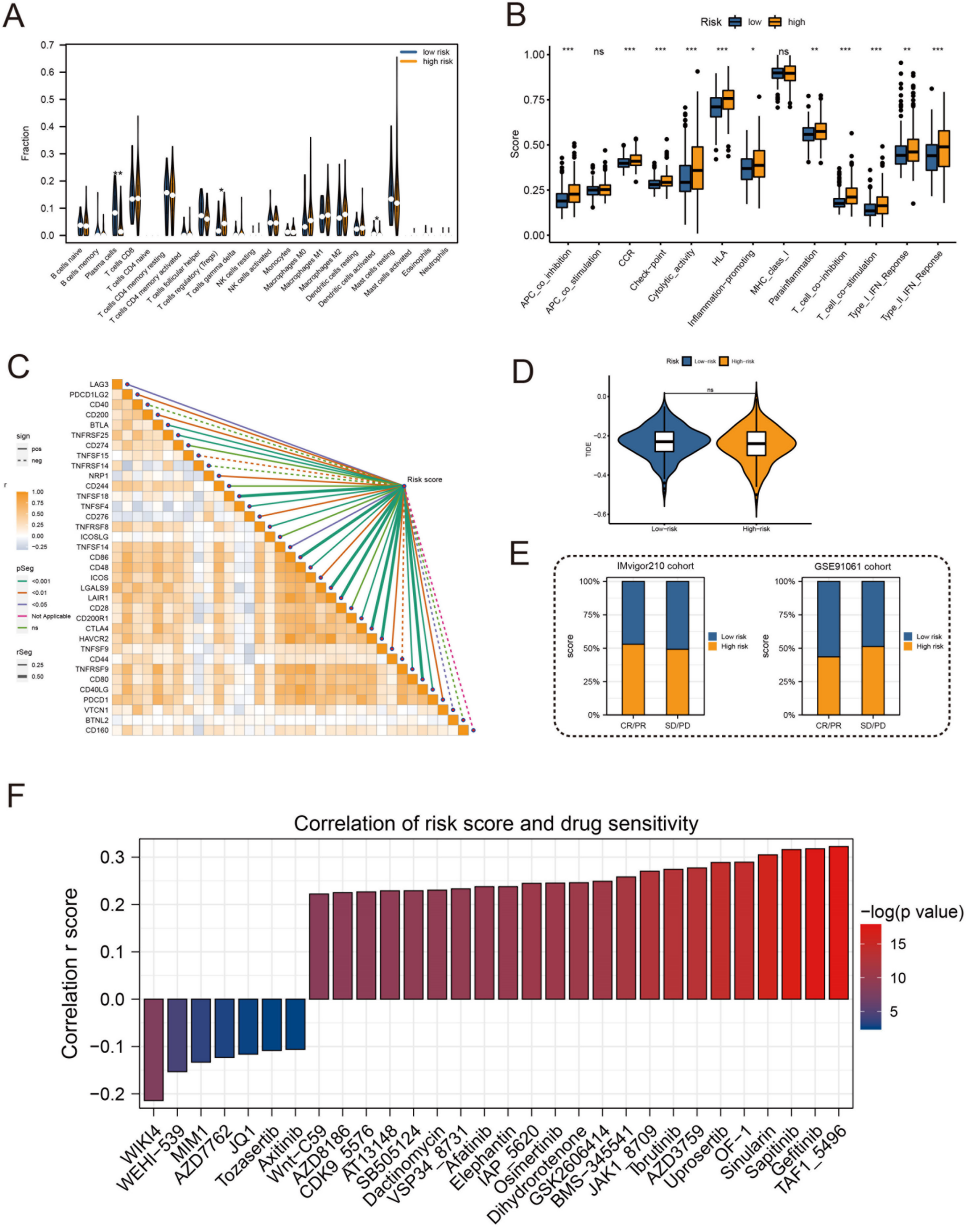
Immune profile and prediction of response to treatment. **(A)** Violin plot for immune cell infiltration analysis. **(B)** Boxplots for immunologic function assays. **(C)** Heatmap of correlation analysis between immunosuppressive receptors and immunosuppressive ligands and risk scores. **(D)** Violin plot for immunotherapy response analysis in high-risk and low-risk groups. **(E)** Bar plots of immunotherapy response analysis for the IMvigor210 cohort and GSE91061 cohort. **(F)** Drug sensitivity analysis between patients in high and low-risk groups. (*:p<0.05, **:p<0.01, ***:p<0.001).

High-risk groups had a higher number of T cell regulatory (Treg) cells than low-risk groups based on immune landscape analysis (**Figure 7A**). Most immune functions increased relatively in the high-risk group (**Figure 7B**). The expression of immunosuppressive receptors and immunosuppressive ligands was also higher in high-risk patients (**Figure 7C**). Additionally, the TIDE algorithm determined that there were no significant differences in immunotherapy response between high-risk and low-risk patients (**Figure 7D**). The prediction results of the IMvigor210 cohort and the GSE91061 cohort showed no difference in the effect of immunotherapy (**Figure 7E**). To evaluate chemotherapy response in PRAD patients with different Anoikis scores, the oncopredict R package was used. Our results showed that the IC50 values of high-risk patients in several chemotherapy molecules were significantly lower, including WIKI4, WEHI−539, MIM1, AZD7762, JQ1, Tozasertib, Axitinib (**Figure 7F**). Overall, immune landscape analysis showed that risk score was associated with different immune responses, and chemotherapy may be more effective than immunotherapy for high-risk patients.

### 2.6 scRNA-seq data analysis

**Figure 8:**
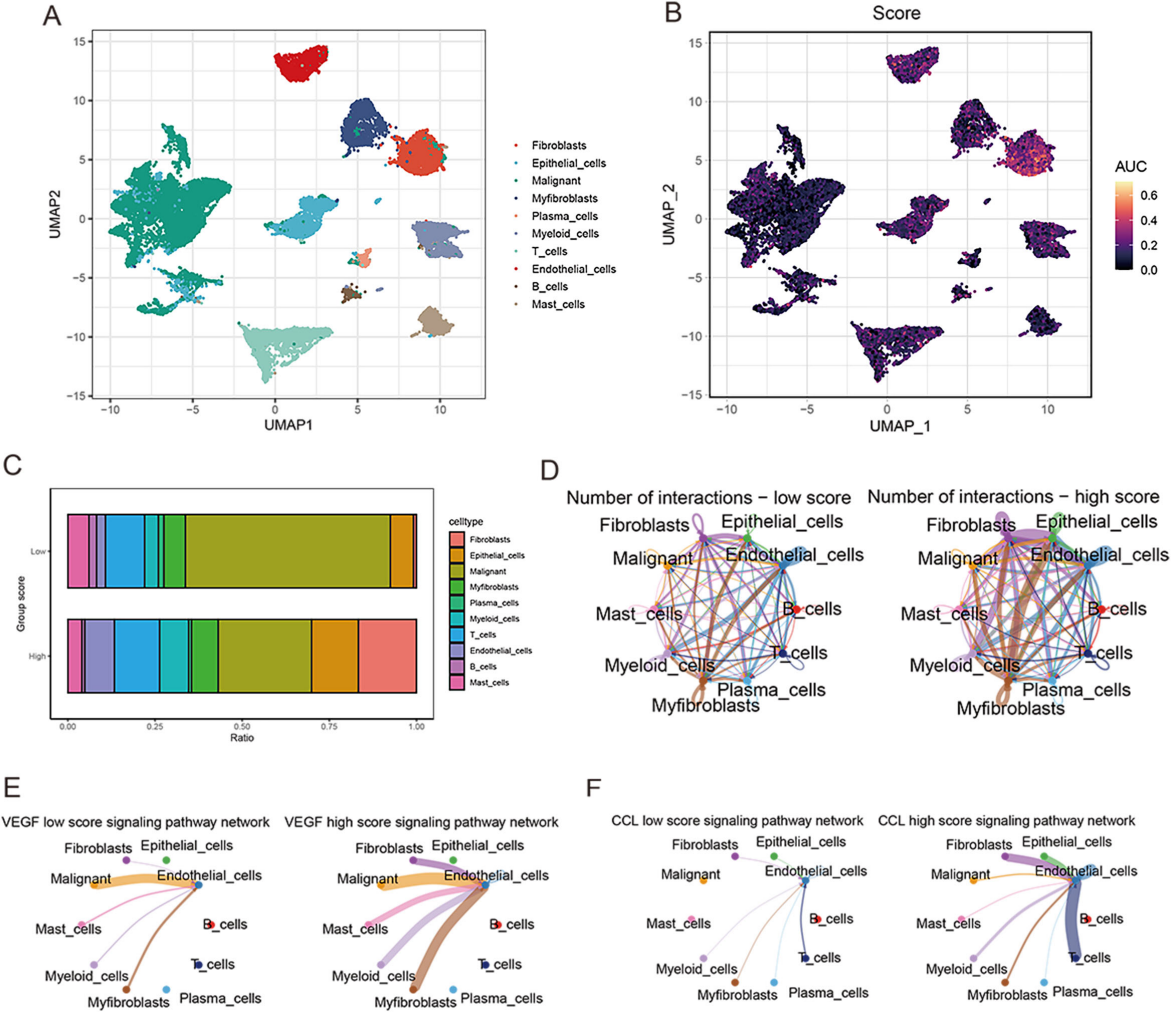
ScRNA-seq data analysis. **(A)** UMAP plot for single-cell dimensional cluster analysis. **(B)** Expression of anoikis score in single cell subsets. **(C)** Bar graph of cell content for samples from the high and low anoikis score groups. **(D)** Network diagram for cellular communication analysis. **(E)** The difference in VEGF signal between high and low anoikis scores. **(F)** The difference in CCL signal between high and low anoikis scores.

After sample pretreatment, the cells were aggregated and annotated into 10 major clusters of fibroblasts, epithelial cells, malignant cells, myofibroblasts, plasma cells, myeloid cells, T cells, endothelial cells, B cells and mast cells (**Figure 8A**). The expression of signature genes in cell subsets suggests that our clustering was successful (**Supplementary Figure S2A**). The distribution of cell types in each sample is shown in **Supplementary Figure S2B**. Subsequently, we divided all cells into high and low groups according to anoikis-related AUC scores (**Figure 8B**). The high anoikis score group showed an increased number and intensity of intercellular interactions based on ligand-receptor signals (**Figures 8C, D**). In comparison with the low anoikis score group, the VEGF signaling pathway network and CCL signaling pathway networks were enhanced in the high anoikis scores group (**Figures 8E, F**). Overall, patients in the high anoikis score group and the low anoikis score group showed differences in intercellular communication, whereas several carcinogenic pathways were significantly overactivated in the high anoikis score group.

### 2.7 Validating the ARG-based signature model genes

**Figure 9:**
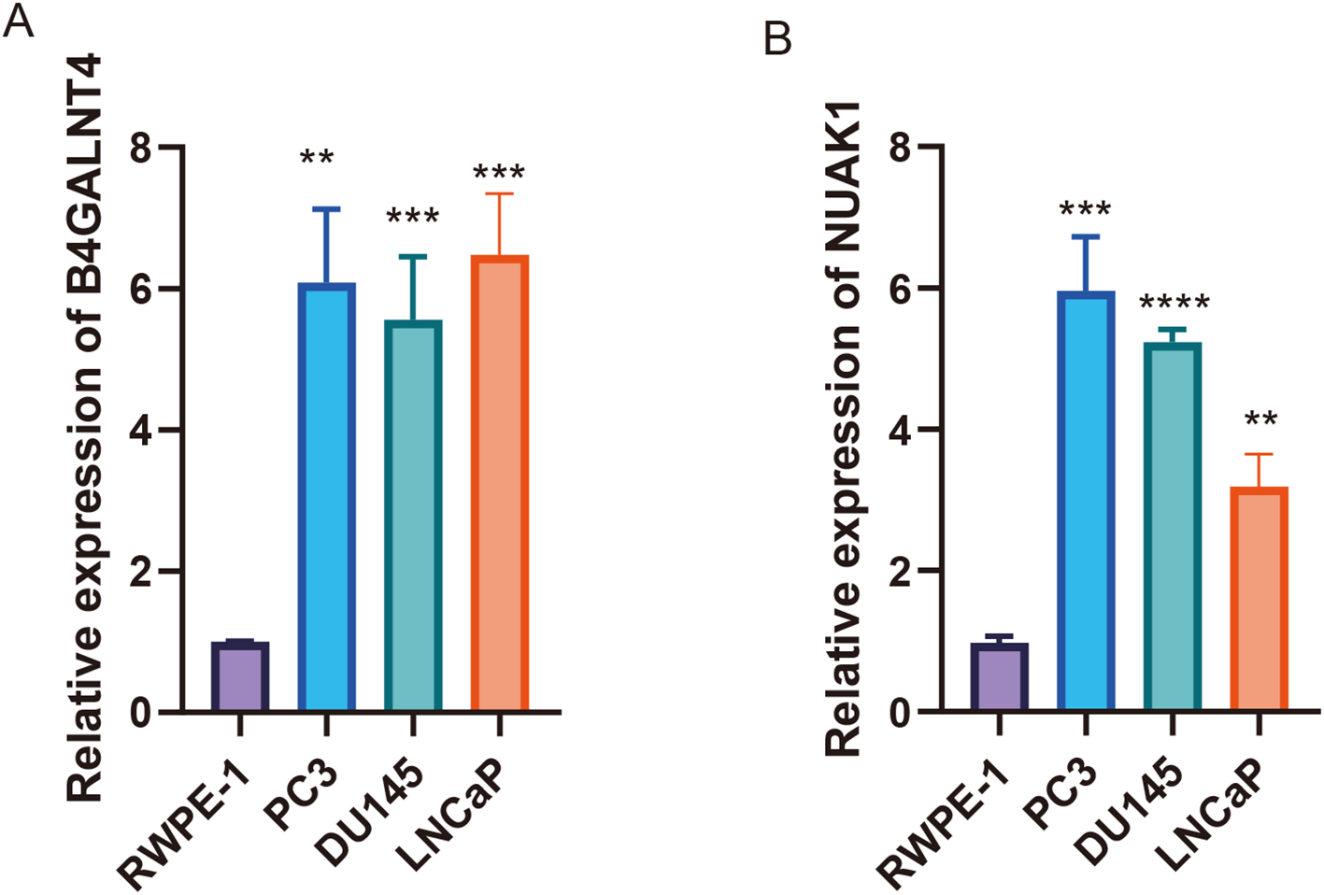
Validating the ARG-based signature model genes. **(A, B)** The expression of B4GALNT4 and NUAK1 in RWPE-1, LNCaP, PC3 and DU145 (**:p<0.01, ***:p<0.001).

To further demonstrate the accuracy of the, we used three prostate cancer cell lines (PC3 (RRID: CVCL_0035), DU145 (RRID: CVCL_0105), and LNCaP (RRID: CVCL_0395)) and a normal prostate epithelial cell line, RWPE-1 (RRID: CVCL_3791). B4GALNT4 and NUAK1 were validated in the model, respectively. the mRNA of B4GALNT4 was sequentially highly expressed in LNCaP, PC3 and DU145, and lowly expressed in RWPE-1 (**Figure 9A**). In addition, the mRNA of NUAK1 was similarly highly expressed in the prostate cancer cell lines (sequentially PC3, DU145, and LNCaP) (**Figure 9B**).

## 3 Discussion

Prostate cancer poses a major threat to men’s health worldwide (18). While prostate-specific antigen (PSA) is widely used for diagnosis and prognostication, it has limitations in accuracy and timeliness. Thus, there is an urgent need for robust biomarkers to improve prediction of prostate cancer prognosis. Recent evidence indicates that apoptosis, a form of programmed cell death, critically regulates the biological behaviors of various cancers (19, 20). For instance, CPT1A which controls fatty acid oxidation can confer anoikis resistance and promote colorectal cancer metastasis (21). IQGAP1 has also been shown to enhance viability and inhibit anoikis by activating Src/FAK signaling in hepatocellular carcinoma, suggesting its potential as a marker for metastasis and prognosis (22). Additionally, CCN2 suppresses lung cancer progression through anoikis pathways involving DAPK (23). Hence, targeting anoikis-related genes may provide promising therapeutic and prognostic opportunities in cancer.

In the present study, we identified a total of 27 anoikis-related genes (ARGs) and developed a robust ARG-based signature model with significant prognostic utility in prostate cancer. This 9-gene model comprised THSD4, PIK3R1, SULF1, B4GALT1, CDC20, COL1A2, S100A10, B4GALNT4 and NUAK1, all of which have established functional relevance in cancer. For instance, THSD4 is downregulated in prostate cancer and cooperates with other genes to drive malignant transformation (24). Clinical sequencing by Chakraborty et al. revealed alterations in PIK3R1 as a potential key regulator of the insulin-PI3K-glycolysis pathway in prostate cancer (25). SULF1 was demonstrated to antagonize Wnt3A-induced growth and disrupt cellular architecture in prostate cancer models (26). B4GALT1 was identified as a unique tumor suppressor silenced by AKR1C3 activation, thereby facilitating castration-resistant prostate cancer progression (27). Additionally, while CDC20, COL1A2 and S100A10 possess recognized pro-oncogenic activities, the precise roles of B4GALNT4 and NUAK1 in prostate cancer warrant elucidation. Functional characterization of these ARGs could unveil novel mechanisms driving disease progression and metastasis. Critically evaluating their clinical utility as prognostic biomarkers and therapeutic targets will enable personalized management. Our findings provide a compelling rationale for investigating this anoikis gene signature, given the predictive power of these 9 ARGs for improving prostate cancer risk assessment, prognostication, and informing clinical decision-making. Future studies validating this signature in independent cohorts and delineating the molecular pathways are warranted to realize its full translational potential.

Through an unbiased gene set variation analysis (GSVA), we identified biological pathways associated with the anoikis gene signature in prostate cancer. Enrichment of established oncogenic pathways including epithelial-mesenchymal transition (EMT), E2F targets, and angiogenesis was observed in the high-risk group, whereas metabolic pathways were downregulated. These pathways have known roles in driving prostate cancer progression. For example, EMT and DNA repair pathway activation can increase therapeutic resistance and invasiveness (27), while E2F inhibition triggers replication stress representing a potential treatment approach (28). Moreover, angiogenesis is a recognized key factor enabling irreversible tumor growth (29, 30). Intriguingly, our GSVA screen also revealed involvement of cholesterol/lipid metabolism and extracellular matrix organization pathways, which have emerging links to prostate cancer through dysregulated lipid metabolism and matrix remodeling (31–33). By systematically delineating the functional interactions between anoikis-related genes and impacted pathways, our findings provide a foundation to uncover novel mechanisms of treatment resistance in prostate cancer. Elucidating how this gene signature influences oncogenic signaling and metabolic programs could illuminate new therapeutic targets and strategies to overcome resistance. Future experimental validation is warranted to realize the full translational potential of these biological insights.

Recently, novel immunotherapeutic approaches have emerged for prostate cancer management (31). The tumor microenvironment comprising stromal cells, vasculature and immune infiltrates plays a crucial role in cancer progression and metastasis (32). Multiple studies have demonstrated that immunosuppressive cells can promote tumor growth and metastasis within the microenvironment (33–35). However, the lack of understanding of the prostate cancer microenvironment and immune landscape has resulted in suboptimal responses to immunotherapy in patients. Additionally, numerous immunotherapies effective in preclinical studies have failed in clinical trials, underscoring the limitations of current prostate cancer models (36). To evaluate the utility of our risk signature in predicting immunotherapy response, we analyzed immune-associated cell infiltration in tumors with high versus low risk scores (37, 38). Our findings suggest this approach of stratifying immunotherapy response holds promise, pending experimental validation. Future research is warranted to systematically characterize the immuno-phenotypes associated with anoikis gene expression, which could guide more precise immunotherapeutic strategies and improve outcomes for prostate cancer patients. Large scale validation studies, especially those incorporating assessments before and after immunotherapy, are essential to firmly establish the clinical utility of this gene signature in immune response prediction.

Although our proposed model demonstrates promising results in predicting prostate cancer prognosis, there remain several limitations that need to be addressed before it can be widely applied in clinical practice (39). Firstly, as the current study utilizes public databases for analysis, the model has not been verified on real-world patient data. Further validation on clinical samples is required to confirm its prognostic power. Secondly, while gene expression profiling can identify potential prognostic biomarkers, additional experiments such as immunohistochemistry, immunofluorescence and analysis of clinical variables are necessary to elucidate the underlying mechanisms and interactions between the identified genes and prostate cancer progression. Thirdly, the potential biological pathways and downstream effects of the prognostic gene signature remain to be fully characterized through *in vitro* and *in vivo* functional studies (40). In addition, we also note the role of epigenetic modifications in prostate cancer, where histone methylation modifications promote epithelial cell migration, proliferation, etc., as well as play a role in the expression of anti-apoptotic genes to enhance the viability of prostate cancer cells. We will consider the more comprehensive role of Anoikis in relation to prostate cancer in future studies (41). Finally, as prostate cancer is a highly heterogeneous disease, the model may need to be optimized and tailored to specific molecular subtypes (42). Extensive analysis on large cohorts reflecting diverse patient populations will help improve its generalizability and clinical utility. In summary, though promising, the current prognostic model requires more rigorous validation and mechanistic investigation before its effects on guiding patient management and improving prostate cancer survival outcomes can be realized. We propose several follow-up studies to address these limitations and bring the model closer to clinical application.

## 4 Materials and methods

### 4.1 Data preprocessing

We downloaded RNA transcriptome data from 501 PRAD tumors and 52 normal tissues in the TCGA database, along with corresponding clinical data. Download standardized RNA expression data and complete clinical data for 231 PRAD patients from the MSKCC database, and 94 PRAD patients from the GEO database. Adjust the batch effect through the ‘sva ‘R package. The IMvigor 210 cohort of bladder cancer patients receiving anti-PD-L1 treatment was obtained through the ‘ IMvigor210 Core Biologies ‘ R package, and the GSE91016 data set receiving anti-PD-1 and anti-CTLA4 treatment was also obtained to predict the efficiency of immunotherapy. In addition, we registered the single-cell RNA sequencing (scRNA-seq) dataset (GSE137829) for six PRAD patients and performed quality control, cell clustering, and annotation using the ‘Seurat ‘R package.

### 4.2 Consensus clustering analysis

From the MSigDB database, 27 anoikis-related genes were identified (**Supplementary Table S1**). The PRAD samples were subdivided according to these genes using the non-negative matrix factorization (NMF) method in the R package ‘ NMF ‘. We used the K-M survival curve to compare biochemical recurrence-free (BCRF) survival between sub-groups. Two gene sets were extracted from the MsigDB database to estimate the difference in biological function and immune activity between subgroups using gene set variation analysis (GSVA) with the ‘ GSVA ‘R package. The statistically significant cut-off for GSVA is p.adjust < 0.05.

### 4.3 Generation of anoikis-related signatures

To establish anoikis-related features, we used WGCNA to find gene modules significantly associated with anoikis-related subgroups and extract corresponding genes. We used the TCGA cohort as the training set, while the MSKCC and GSE70769 datasets were the validation sets. A univariate Cox analysis was performed to examine prognostic genes (p<0.05). Using the ‘ randomForestSRC ‘ R package, the prognostic genome was further reduced using Random Survival Forests (RSF). A smaller value indicates greater predictability when variables were sorted by minimum depth in RSF analysis. Using multivariate Cox regression analysis, the best features associated with anoikis were identified based on their respective coefficients (β) and gene expression levels (Exp). The formula is used to calculate each patient’s anoikis-related risk score. Using the median of their anoikis scores, we further categorize the patients into two groups. Kaplan-Meanoikis was used to determine prognostic differences between the two groups. In addition, we examined the correlation between anoikis score and clinical features, including age, PSA level, TN stage, and Gleason score (GS). Cox analyses were performed univariately and multivariate to evaluate the prognostic significance of Anoikis scores. Similarly, we collected the MSKCC and GSE70769 cohorts to check the risk score’s predictive ability.

### 4.4 Functional enrichment analysis

Genes that are differentially expressed between low-risk and high-risk cohorts have been identified as potential mechanisms behind anoikis. Gene Ontology (GO) enrichment and Kyoto Encyclopedia of Genes and Genomes (KEGG) pathway analysis were performed using the R package clustanoikisProfilanoikis. The R package ‘ loop ‘ shows GO and KEGG terms with p 0.05. The MSigDB was analyzed using GSVA to determine the differences in the carcinogenic marker pathways (h.all v7.1.symbols) between the two cohorts. For the same signature pathway, gene set enrichment analysis (GSEA) was conducted using the ‘GSEA ‘R package (FDR < 0.25, NES > 1, p.adjust < 0.05). The prognostic significance of GSVA and GSEA overlapping marker pathways was determined using the K-M method.

### 4.5 Somatic mutation analysis

Somatic mutations in PRAD patients were extracted from the TCGA database. The ‘ maftools ‘ R package explored specific somatic mutation variants in different risk score groups. Next, we studied the coexistence or exclusion of mutations, oncogenes, and enrichment of known carcinogenic pathways between the two cohorts. The tumor mutation burden (TMB) reflecting the total mutation count of each PRAD patient was calculated and its correlation with the anoikis score was tested. In addition, we analyzed the predictive value of TMB and Anoikis score for survival outcomes in the Anoikis score risk cohort.

### 4.6 Immune landscape and treatment response prediction

In high-risk and low-risk groups, we compared immune cell abundance, immune function, and immune checkpoints. On the basis of RNA expression profiles of PRAD patients, the tumor immune dysfunction and rejection algorithm (TIDE) predicts potential immunotherapy responses. The IMvigor210 and GSE91061 datasets were also used to determine the correlation between the Anoikis score and the efficacy of potential immunotherapy. In addition, we investigated the chemotherapy responses of the two groups of patients, and the ‘ oncopredict ‘ R package predicted the sensitivity of each patient to chemotherapy.

### 4.7 scRNA-seq data analysis

Next, we use the GSE137829 dataset to study the single-cell characteristics of PRAD. The software Seurat (version 4.3.0) were then used to process and evaluate the gene expression matrix. Based on the number of identified genes per cell (500–7000) and the percentage of mitochondrial genes expressed (10%), we performed Seurat-based filtering of the cells. Additionally, the ribosomal and mitochondrial genes were taken out of the gene expression matrix. After quality inspection, 21,292 high-quality cells with an average of 2419 genes per cell were kept. Then, we calculated the activity of risk score-related gene sets at the single cell level through the ‘ AUCell ‘R package. After dividing all cells according to AUC, we classified them into two groups: high and low. By using the R software package CellChat, signaling pathways were analyzed between participants with high anoikis scores and those with low anoikis scores.

### 4.8 qRT-PCR

Prostate cancer cell lines (PC3 (RRID: CVCL_0035), DU145 (RRID: CVCL_0105) and LNCaP (RRID: CVCL_0395)) and normal prostate epithelial cell line RWPE-1 (RRID: CVCL_3791) were purchased from Shanghai Zhongqiao Xinzhou Biotech Co. and cultured in DMEM medium containing 10% FBS and 1% penicillin-streptomycin (Solarbio, Beijing, China). FBS and 1% penicillin-streptomycin in DMEM medium (Solarbio, Beijing, China). For isolation of total RNA, TRIzol reagent (from Invitrogen, Carlsbad, CA, USA) was used and RNA was reverse transcribed to cDNA using ReverTra Ace qPCR RT premix and gDNA Remover kit. cDNA was extracted from the RNA by SYBR Premix Ex Taq II on a Mx3005P Real-Time Fluorescence Quantitative PCR System (from Stratagene, San Diego, CA, USA). qRT-PCR was performed and GAPDH was selected as an endogenous control for mRNA. The reaction conditions were pre-denaturation at 95°C for 10 min, denaturation at 95°C for 5 s, and annealing at 60°C for 30 s, for a total of 45 cycles. Amplification of target and internal endogenous reference genes was performed separately for each sample. Each set of samples contained 3 replicate wells. Data were analyzed using the 2^(-ΔΔCt) method. The primer sequences are detailed in **Supplementary File 1**.

## 5 Conclusions

This comprehensive study unravels the intricate relationship between anoikis and bone metastasis in prostate cancer. Our findings shed light on the critical role of anoikis in driving metastatic progression, contributing to our understanding of the underlying biomarkers and molecular mechanisms. The identified anoikis-related gene signatures and dysregulated molecular pathways hold promise as potential targets for prognostication and therapeutic interventions in the management of prostate cancer.

## Data Availability

The original contributions presented in the study are included in the article/**Supplementary Material**. Further inquiries can be directed to the corresponding author.

## Supplementary material

Supplementary Figure 1 The optimal number of clusters was determined by co-occurrence, dispersion, and contour indices, and the optimal number of clusters selected was 2.

Supplementary Figure 2 Single cell dimension reduction cluster analysis. **(A)** Bubble plots showing expression of signature genes in cell subsets. **(B)** Distribution of cell types in each sample.
