# Supplementary material for: Anoikis in prostate cancer bone metastasis gene signatures and therapeutic implications
Source: Front Oncol. 2024 Sep 26;14:1446894. doi: 10.3389/fonc.2024.1446894 (PMC11464922; doi:10.3389/fonc.2024.1446894)
Supplement: Supplementary file 1 [file Table1.docx]

**Supplementary Table S1. Summary of anoikis-related genes**

| **Gene** | **Type** |
| --- | --- |
| **CEACAM5** | **Anoikis** |
| **PTK2** | **Anoikis** |
| **PIK3CA** | **Anoikis** |
| **CEACAM6** | **Anoikis** |
| **PDK4** | **Anoikis** |
| **BCL2** | **Anoikis** |
| **TSC2** | **Anoikis** |
| **PTRH2** | **Anoikis** |
| **STK11** | **Anoikis** |
| **MAP3K7** | **Anoikis** |
| **NTRK2** | **Anoikis** |
| **CRYBA1** | **Anoikis** |
| **MCL1** | **Anoikis** |
| **DAPK2** | **Anoikis** |
| **NOTCH1** | **Anoikis** |
| **TLE1** | **Anoikis** |
| **CAV1** | **Anoikis** |
| **SNAl2** | **Anoikis** |
| **IKBKG** | **Anoikis** |
| **SRC** | **Anoikis** |
| **AKT1** | **Anoikis** |
| **MTOR** | **Anoikis** |
| **CHEK2** | **Anoikis** |
| **E2F1** | **Anoikis** |
| **ITGA5** | **Anoikis** |
| **ITGB1** | **Anoikis** |
| **BRMS1** | **Anoikis** |
