# Supplementary figures and images for: Anoikis in prostate cancer bone metastasis gene signatures and therapeutic implications
Source: Front Oncol. 2024 Sep 26;14:1446894. doi: 10.3389/fonc.2024.1446894 (PMC11464922; doi:10.3389/fonc.2024.1446894)

# NMF rank survey

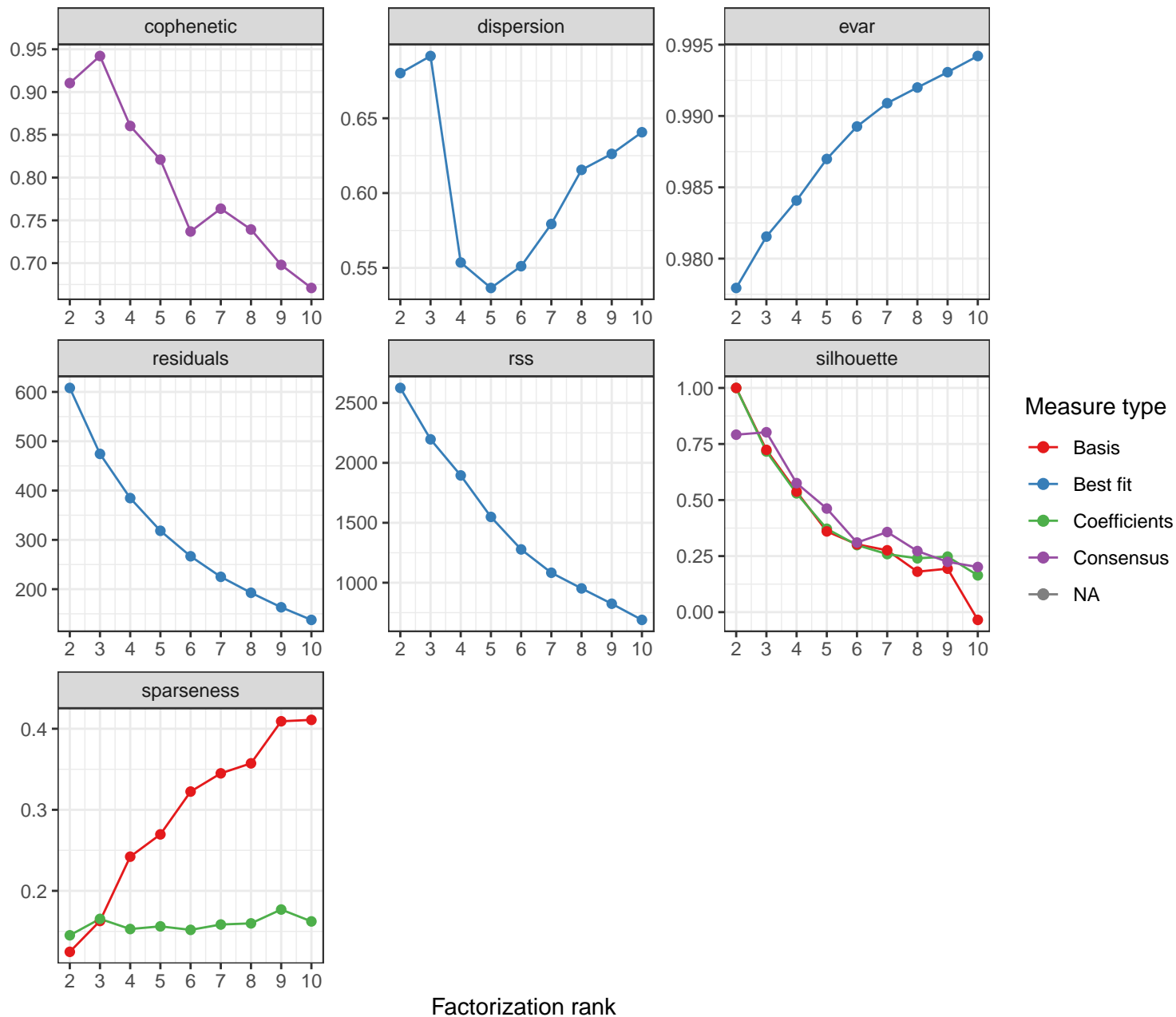

Supplement: Supplementary Figure 1 — The optimal number of clusters was determined by co-occurrence, dispersion, and contour indices, and the optimal number of clusters selected was 2. [file DataSheet1.pdf]

identity

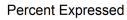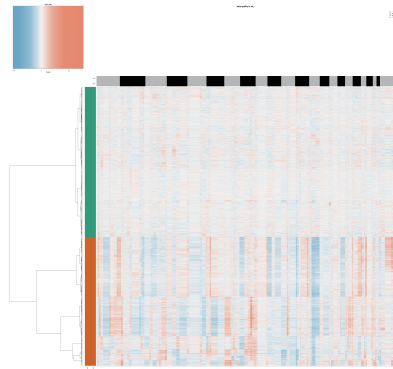

UMAP2

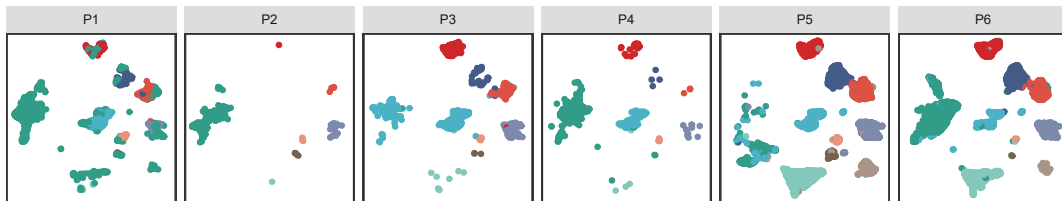

UMAP1

Supplement: Supplementary Figure 2 — Single cell dimension reduction cluster analysis. (A) Bubble plots showing expression of signature genes in cell subsets. (B) Distribution of cell types in each sample. [file DataSheet2.pdf]
